# Knockdown of LAP2α inhibits osteogenic differentiation of human adipose-derived stem cells by activating NF-κB

**DOI:** 10.1186/s13287-020-01774-9

**Published:** 2020-07-01

**Authors:** Yiman Tang, Xiao Zhang, Wenshu Ge, Yongsheng Zhou

**Affiliations:** 1 Department of Prosthodontics, Peking University School and Hospital of Stomatology, Beijing, China; 2 Fourth Clinical Division, Peking University School and Hospital of Stomatology, Beijing, China; 3 National Clinical Research Center for Oral Diseases, National Engineering Laboratory for Digital and Material Technology of Stomatology, Beijing Key Laboratory of Digital Stomatology, Beijing, China; 4 Department of General Dentistry II, Peking University School and Hospital of Stomatology, 22 Zhongguancun Avenue South, Haidian District, Beijing, China

**Keywords:** LAP2α, Nuclear factor-κB, Osteogenic differentiation, Human adipose-derived stem cells

## Abstract

**Background:**

Lamina-associated polypeptide 2α (LAP2α) is a nucleoplasmic protein that has been involved in the regulation of the cell cycle, gene transcription, and adult stem cell function. LAP2α down-regulation is linked to age-related osteoporosis and bone deformities; however, the underlying mechanisms remain obscure. The present study aimed to elucidate the function of LAP2α in the osteogenic differentiation of human adipose-derived stem cells (hASCs), which are attractive sources for bone tissue engineering.

**Methods:**

The expression of LAP2α during the osteogenic differentiation of hASCs was detected firstly. A loss of function investigation was then carried out to characterize the function of LAP2α in osteogenic differentiation of hASCs both in vitro and in vivo. Moreover, RNA-sequences, western blotting, and confocal analyses were performed to clarify the molecular mechanism of LAP2α-regulated osteogenesis.

**Results:**

We found that LAP2α expression was upregulated upon osteogenic induction. Both in vitro and in vivo experiments indicated that LAP2α knockdown resulted in impaired osteogenic differentiation of hASCs. Mechanistically, we revealed that LAP2α deficiency activated nuclear factor kappa B (NF-κB) signaling by controlling the cytoplasmic-nuclear translocation of p65.

**Conclusions:**

Collectively, our findings revealed that LAP2α functions as an essential regulator for osteogenesis of hASCs by modulating NF-κB signaling, thus providing novel insights for mesenchymal stem cell-mediated bone tissue engineering.

## Background

Adult mesenchymal stem cells (MSCs), such as human adipose-derived stem cells (hASCs), can differentiate into multiple cell lineages and show the capacity for self-renewal. hASCs are well accepted as ideal seed cells for bone regeneration [1, 2]. The cell fate commitment of hASCs is considered to be a complex process regulated by the orchestrated activation or repression of lineage-specific genes. Multiple signaling pathways, including those of bone morphogenetic protein (BMP)/Smad, nuclear factor-κB (NF-κB), Wnt/β-catenin, Hedgehog, and PTH/PTHrP, are involved in the regulation of osteogenic differentiation [3–6]. Therefore, it is pivotal to better understand the molecular mechanisms of the osteogenesis of hASCs to promote the development of hASC-based clinical applications.

Lamina-associated polypeptide 2α (LAP2α), encoded by the *TMPO* gene (Thymopoietin), an exclusively mammalian non-membrane-associated isoform of LAP2, is localized throughout the nuclear interior, where its N-terminal common domain interacts with chromatin [7, 8]. Inside the nucleus, the unique C-terminal α-specific region of LAP2α binds a particular subset of A-type lamins and regulates cell cycle progression via retinoblastoma (Rb)-mediated E2F-dependent transcription [9, 10]. Previous studies have shown that loss of LAP2α in mice leads to selective depletion of the nucleoplasmic lamin A pool, resulting in certain tissue-specific phenotypes, such as disrupted heart function, delayed skeletal muscle differentiation, and enhanced proliferation of tissue progenitor cells in the epidermis, colon, and the hematopoietic system [11–13]. Moreover, LAP2α downregulation is linked to one of the typical cellular phenotype of the Hutchinson-Gilford progeria syndrome (HGPS), manifested by accelerated aging, lipodystrophy, and accelerated bone loss [14, 15]. Thus, LAP2α functions as one of the key regulators of tissue progenitor cell differentiation, suggesting that it might be involved in tissue homeostasis by regulating the balance between differentiation and proliferation of adult stem cells. However, little is known regarding the roles of LAP2α in fate determination or orientated differentiation of MSCs.

The master transcription factor, NF-κB, is involved in many cellular processes, including inflammatory response, immune response, cell apoptosis, and differentiation [16]. Growing evidences indicate that activation of NF-κB signaling impairs both the osteogenesis capability of MSCs and osteoblast-mediated bone formation [17, 18]. Moreover, selective targeting of NF-κB signaling could block the receptor activator for nuclear factor κB ligand (RANKL)-induced osteoclastogenesis and prevent osteoporotic bone destruction [19]. In addition, several researchers also demonstrated that estrogen deficiency activates NF-κB pathway, resulting in the inhibition of the odonto/osteogenic differentiation of dental pulp stem cells [20, 21]. Thus, the wide involvement of NF-κB in skeletal remodeling and bone homeostasis makes it as a potential therapeutic target to inhibit bone resorption and promote bone formation. In addition, factors affecting NF-κB expression or transcriptional activity might also be important in osteogenic differentiation.

The present study aimed to elucidate the biological and functional roles of LAP2α in hASCs osteogenic differentiation. Our results demonstrated that knockdown of LAP2α inhibited the osteogenic differentiation of hASCs, not only in vitro but also in vivo. Mechanistically, we revealed that LAP2α deficiency activated NF-κB signaling by controlling the cytoplasmic-nuclear translocation of p65, suggesting the potential utility of LAP2α in hASCs-based bone tissue engineering.

## Methods

### Cell culture

The primary hMSCs used in the experiments were purchased from ScienCell Research Laboratories (San Diego, CA, USA), including hASCs (human adipose-derived stem cells) and hBMMSCs (human bone marrow-derived mesenchymal stem cells). The catalog number and lot number of the cells are hASCs (Cat. No. 7510) from three donors with different lot number (Lot. No. 19382, Lot. No. 11537, Lot. No. 8278); hBMMSCs (Cat. No. 7500) from two donors with different lot number (Lot. No. 21580 and Lot. No. 15901). Cells were used to perform the in vitro experiments repeated for three times. Laboratory reagents and materials were obtained from Sigma-Aldrich (St. Louis, MO, USA) unless stated otherwise. Proliferation media (PM) comprising Dulbecco’s modified Eagle’s medium (DMEM) (Gibco, Grand Island, NY, USA) with 10% (v/v) fetal bovine serum (ScienCell) and 1% (v/v) antibiotics (Gibco) was used for cell culture. Osteogenic differentiation was induced after the cells reached 70–80% confluence using osteogenic media (OM), containing standard PM supplemented with 10 mM β-glycerophosphate, 100 nM dexamethasone, and 0.2 mM ascorbic acid.

### Lentivirus infection and establishment of stably transfected cell lines

GenePharma Company (Shanghai, China) provided recombinant lentiviruses targeting LAP2α (shLAP2α-1 and shLAP2α-2) and the scramble control (shNC). The shRNA target sequences comprised shNC, TTCTCCGAACGTGTCACGT; shLAP2α-1, GTCTGTATAAAGCAGTGTA; and shLAP2α-2, GTCTGTATAAAGCAGTGTA. All lentivirus vectors contained puromycin-resistance and green fluorescent protein gene (GFP). According to the manufacturer’s instruction, transfection of cells was performed by exposing them to the viral supernatants combined with polybrene (5 μg/ml) for 24 h. Stably transfected cells were selected using puromycin at 1 μg/ml after transfection for 72 h. The proportion of GFP-positive cells was tracked under an inverted fluorescence microscope (TE2000-U; Nikon) to verify the transduction efficiency.

### Alkaline phosphatase (ALP) staining and quantification

After osteogenic induction for 7 days, cells were subjected to ALP staining and quantification as previously described [22]. ALP staining was conducted using the NBT/BCIP staining kit (CoWin Biotech, Beijng, China). An ALP assay kit (Nanjing Jiancheng Bioengineering Institute, Nanjing, China) was used to quantify ALP activity. The bicinchoninic acid (BCA) method with a Pierce protein assay kit (Thermo Scientific, Rockford, IL, USA) was used to measure the total protein concentration. After normalization to the total protein concentration, the ALP levels relative to the control group were analyzed.

### Alizarin red S (ARS) staining and quantification

After osteogenic induction for 14 days, cells were subjected to matrix mineralization as previously described [23]. Briefly, the cells were fixed for 30 min in 95% ethanol before being stained for 10 min with Alizarin red S (0.1%, pH 4.2). Calcium deposition was assessed quantitatively by eluting ARS with 100 mM cetylpyridinium chloride for 1 h and then detected by spectrophotometric absorbance at 562 nm.

### Immunofluorescence staining

Cells were seeded onto glass coverslips on 12-well plates. To avoid the GFP interference in the stably transfected cell lines, cells transfected transiently by small interfering RNA (siRNA) without fluorescent were used to conduct the immunofluorescence staining. The cells were fixed using 4% paraformaldehyde and subjected to 0.1% Triton X-100 permeabilization for 10 min, before being blocked with 0.8% bovine serum albumin for 1 h. The cells were then incubated with primary antibody (diluted 1:200) recognizing p65 (Cell Signaling Technology) and LAP2α (Abcam, Cambridge, MA, USA) at 4 °C overnight, before being incubated with corresponding secondary antibodies for 1 h at room temperature. DAPI (2-(4-amidinophenyl)-1H-indole-6-carboxamidine) was used to counterstain nuclei. A Confocal Zeiss Axiovert 650 microscope was used to view the cells on the coverslips at 488 nm (green, P65), 549 nm (red, LAP2α), and 405 nm (blue, DAPI). Quantification of p65 nuclear translocation was performed using immunofluorescence microscopy and the public domain image analysis software ImageJ according to Noursadeghi M’s study [24].

### Quantitative reverse transcription-PCR (qRT-PCR)

The Trizol reagent (Invitrogen, Carlsbad, CA, USA) was used to isolate total cellular RNA, which was subsequently subjected to reverse-transcription into cDNA using a PrimeScript RT Reagent Kit (Takara, Kusatsu, Shiga, Japan) as described previously [25]. Quantitative PCR was conducted on an ABI 7500 Real-Time PCR Detection System (Applied Biosystems, Foster City, CA, USA). GAPDH was used as the internal mRNA standard. The primer sequences were as follows: *GAPDH*, (forward) 5′-GGAGCGAGATCCCTCCAAAAT-3′ and (reverse) 5′-GGCTGTTGTCATACTTCTCATGG-3′; *LAP2α* (forward) 5′- ACAGTGACAATGAAGAAGGAAAGA − 3′ and (reverse) 5′- AGGAAAAGAAATACCCTGAAAAAA-3′; *RUNX2*, (forward) 5′-CCGCCTCAGTGATTTAGGGC-3′ and (reverse) 5′-GGGTCTGTAATCTGACTCTGTCC-3′; *ALP*, (forward) 5′-ATGGGATGGGTGTCTCCACA-3′ and (reverse) 5′-CCACGAAGGGGAACTTGTC-3′; *OCN*, (forward) 5′-AGCAAAGGTGCAGCCTTTGT-3′ and (reverse) 5′-GCGCCTGGGTCTCTTCACT-3′; *IL6*, (forward) 5′- CGCAACAACTCATCTCATTCTGCG-3′ and (reverse) 5′- CATGCTACATTTGCCGAAGAGC-3′; *ICAM1*, (forward) 5′-AGTGTGACCGCAGAGGACGA-3′ and (reverse) 5′-GGCGCCGGAAAGCTGTAGAT-3′; *TRAF1*, (forward) 5′-CGGTGCTCTTGATCCCTACTCACCG-3′ and (reverse) 5′-GAATGGCTGCATCTCATGCTCT-3′. The 2^-△△Ct^ method was used to analyze the data.

### Western blotting

Western blotting analysis was performed as previously described [26]. Briefly, the cells were lysed in RIPA buffer, followed by sonication and centrifugation to get suspensions. Ten percent of sodium dodecylsulfate-polyacrylamide gel electrophoresis (SDS-PAGE) was used to separate the proteins of different molecular weight, which was transferred electrophoretically to polyvinylidene difluoride (PVDF) membranes. The membranes were blocked and then incubated with primary antibodies recognizing GAPDH, RUNX2, p-p65 (Ser536), p-IκBα (ser32/ser36), and p65 (Cell Signaling Technology), and LAP2α (Abcam, Cambridge, MA, USA) at 4 °C overnight. After washing, the membranes were then incubated with the appropriately-labeled secondary antibodies for 1 h at room temperature. Immunoreactive protein bands were detected with an ECL kit (CWBIO, Beijing, China).

### RNA-seq analysis

Total RNA was extracted from hASCs transfected with shLAP2α and shNC. RNA sequencing of the control and LAP2α knockdown cells was conducted using BGISEQ-500 sequencing system [27]. Differentially expressed genes (DEGs) between the control and LAP2α knockdown cells were picked out via the NOISeq method [28]. To identify the DEGs, the fold differences (≥ 2), probability (≥ 0.8), and their average expression were used. KEGG pathway enrichment analysis and Gene Ontology analysis of DEGs were subsequently performed [29, 30]. The RNA-seq data can be obtained in the Gene Expression Omnibus (GEO) database at the National Center for Biotechnology Information (accession number GEO: GSE138512).

### Heterotopic bone formation in vivo

The Peking University Animal Care and Use Committee approved the in vivo study (LA2019019). The in vivo study was conducted as previously described [31]. For 7 days before the implantation study, hASCs infected with lentivirus harboring shNC, shLAP2α-1, and shLAP2α-2 were induced into osteoblasts. Beta-tricalcium phosphate (β-TCP) bone graft (Bicon, Boston, MA, USA) was then incubated with the cells for 1 h at 37 °C to form scaffolds. Thereafter, the mixtures were transplanted subcutaneously symmetrically into two sites on the dorsal regions of 6-week-old BALB/c homozygous nude (nu/nu) mice, and the scaffolds containing hASCs/shNC, hASCs/shLAP2α-1, and hASCs/shLAP2α-2 were randomly transplanted into three groups of mice (*n* = 8 per group). Eight weeks after surgery, specimens were harvested and fixed in 4% paraformaldehyde for further experiments.

### H&E staining, Masson’s trichrome, and immunohistochemical analysis

Specimens were subjected to decalcification in 10% EDTA (pH 7.4) for 2 weeks, before being embedded in paraffin. Paraffin sections with 4-mm thickness were stained using H&E and Masson’s trichrome. To evaluate osteogenesis, osteocalcin was evaluated using immunohistochemistry. The histological specimens were viewed using a light microscope (Olympus, Tokyo, Japan). For quantification of bone-like tissue, 2 images of each sample (16 images for each group) were taken randomly. Image-Pro Plus software (Media Cybernetics, Rockville, MD, USA) was used to evaluate the proportion of new osteoid area [(bone area/total tissue area) × 100%] or mean density (integrated optical density of positive staining/total tissue area) of immunohistochemical staining. The quantitative results were shown in box-plot.

### Cell viability assays

The cell viability was evaluated with a Cell Counting Kit-8 (CCK8) (Dojindo, Kumamoto, Japan). Cells were seeded at 5 × 10^3^ cells per well in 48-well plates and cultured in proliferation medium. At each time point, the supernatant of each group was removed, and cells were incubated with DMEM medium containing CCK-8 for 2 h at 37 °C. Optical density (OD) was measured at 450 nm using a microplate reader (ELX808, BioTek).

### Statistical analysis

All statistical calculations were performed using SPSS 20.0 software (IBM Corp., Armonk, NY, USA). Data from at least three independent experiments are shown as the mean ± the standard deviation (SD). The Shapiro–Wilk test served to test the normal distribution of the variables. Differences between two groups were analyzed by Student’s independent *t* test, while differences between multiple groups were analyzed by one-way analysis of variance (ANOVA) followed by Bonferroni correction. The *P* values were considered statistically significant if less than 0.05.

## Results

### LAP2α is upregulated in hASCs upon osteogenic differentiation

**Fig. 1:**
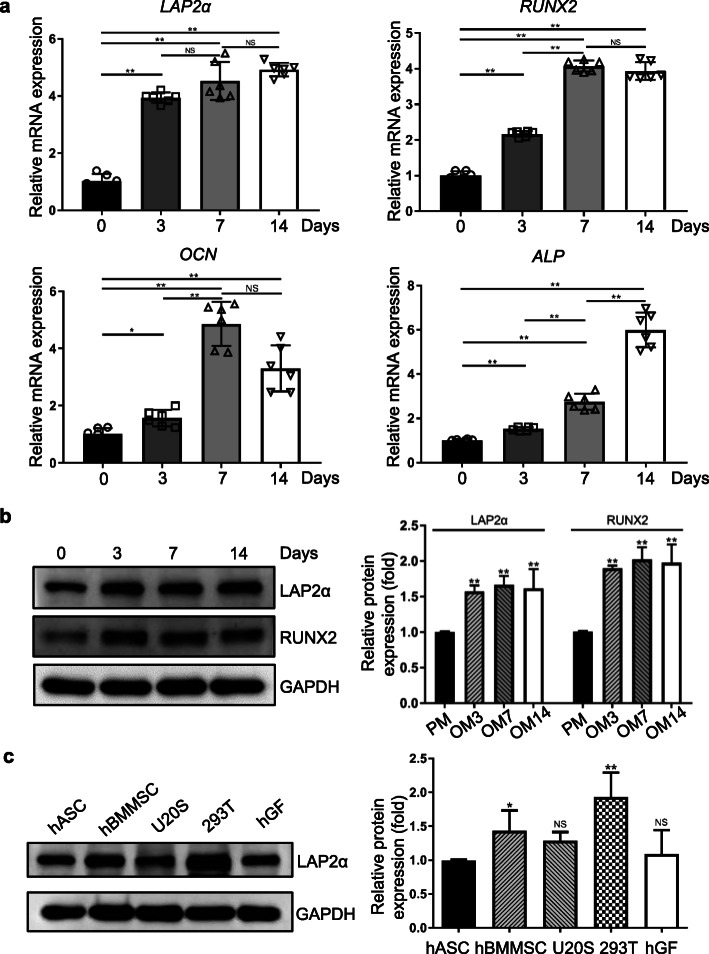
LAP2α is upregulated in hASCs upon osteogenic differentiation. **a** Relative mRNA expression of *LAP2α* and the osteogenic markers *RUNX2*, *ALP*, and *OCN*, as measured by qRT-PCR during osteogenic differentiation of hASCs. *GAPDH* was used for normalization, *n* = 6. **b** Western blotting and quantification of protein levels of LAP2α, RUNX2, and the internal control GAPDH during osteogenic differentiation of hASCs, *n* = 3. **c** Western blotting and quantification of protein levels of LAP2α in hASCs, hBMMSCs, osteosarcoma cell lines U20S, 293T, and human gingival fibroblasts (hGF), *n* = 3. Data are shown as the mean ± SD; *P < 0.05 compared with the control group; **P < 0.01 compared with the control group; NS: not significant

In order to identify the potential role of LAP2α in osteogenesis, we first examined the expression of LAP2α in hASCs after osteoblastic induction. As shown in Fig. 1a, quantitative reverse transcription-PCR (qRT-PCR) revealed marked upregulation of the endogenous expression level of *TMPO* in hASCs within 14 days of osteoblastic induction. The mRNA expression levels of osteogenic markers *RUNX2* (encoding RUNX family transcription factor 2), *ALP* (encoding alkaline phosphatase), and *OCN* (encoding osteocalcin) were also elevated during osteogenic differentiation. Similar results were observed for the dynamic expression profile of these genes, as monitored by western blotting analysis (Fig. 1b). These results implied that LAP2α is potentially associated with the osteogenic differentiation of hASCs. Moreover, we compared the LAP2α expression in hASCs, hBMMSCs, osteosarcoma cell lines U2OS, human embryonic kidney cells 293T, and human gingival fibroblasts. As shown in Fig. 1c, 293T cells had the highest levels of LAP2α among the five groups. The protein level of LAP2α was significantly higher in hBMMSCs than in hASCs, while there was no significant difference among hASCs, U2OS, and human gingival fibroblasts.

### LAP2α is required for osteogenic differentiation of human mesenchymal stem cells in vitro

**Fig. 2:**
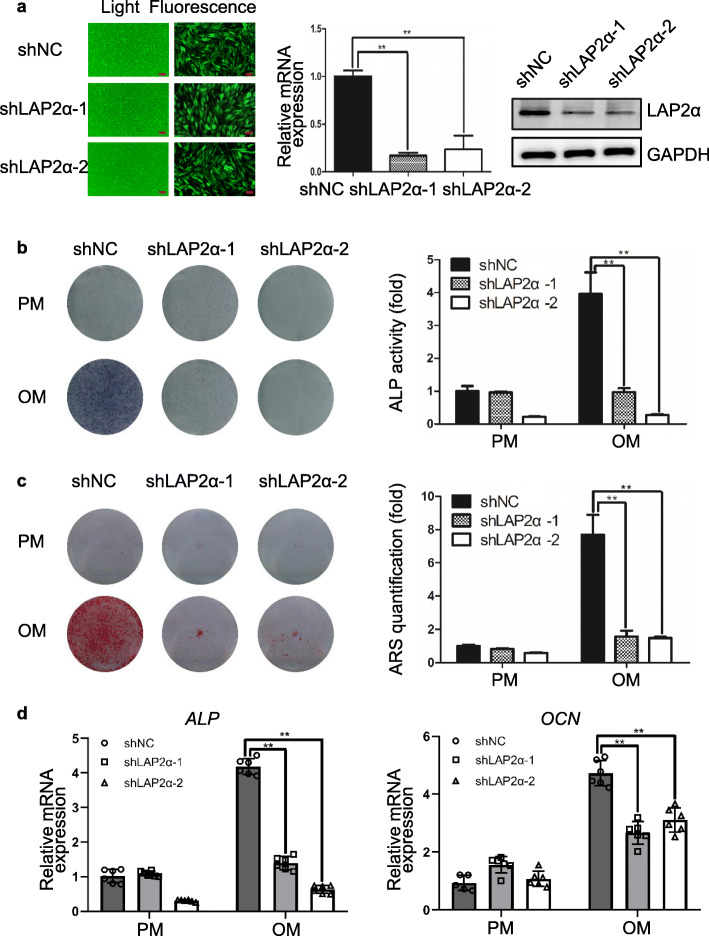
Knockdown of LAP2α inhibits osteogenic differentiation of hASCs in vitro. **a** Left panel: fluorescent micrographs showing the efficiency of lentivirus transfection. Scale bars: 100 μm. Middle and right panel: Validation of LAP2α knockdown effect by qRT-PCR and western blotting, respectively, *n* = 3. **b** ALP staining and quantification of cells in the shNC, shLAP2α-1, and shLAP2α-2 groups on day 7 after osteogenic induction, *n* = 3. **c** ARS staining and quantification of cells in the shNC, shLAP2α-1, and shLAP2α-2 groups on day 14 after osteogenic induction, *n* = 3. **d** Relative mRNA expression of *ALP* and *OCN*, as measured by qRT-PCR on day 14 of osteogenic induction. *GAPDH* was used for normalization, *n* = 6. Data are shown as the mean ± SD; *P < 0.05 compared with the control group; **P < 0.01 compared with the control group; NS: not significant

To investigate the role of LAP2α in osteogenic differentiation of hASCs, we first constructed hASCs stably depleted for LAP2α expression using a lentiviral-driven knockdown strategy. To avoid possible off-target effects, two different sets of short hairpin RNAs (shRNAs) targeting different regions of LAP2α were designed. The efficiency of lentiviral transduction was more than 80%, as determined by fluorescent microscopy (Fig. 2a and Additional file 1: Fig. S1a). The knockdown efficiency was assessed by qRT-PCR, western blotting, and immunofluorescence assay (Fig. 2a and Additional file 1: Fig. S1b-c). Meanwhile, we observed that LAP2α depletion had a marginal effect on the proliferation of hASCs (Additional file 2: Fig. S2). After osteoblastic induction, ALP activity was markedly inhibited in LAP2α knockdown hASCs (Fig. 2b and Additional file 3: Fig. S3a). Meanwhile, extracellular matrix mineralization, as assessed by ARS staining and quantification, was also significantly inhibited in LAP2α-depleted cells (Fig. 2c and Additional file 3: Fig. S3b). Moreover, qRT-PCR analysis indicated that the expression levels of osteogenic markers such as *ALP* and *OCN* were significantly attenuated upon LAP2α knockdown (Fig. 2d). Collectively, these results demonstrated that LAP2α knockdown inhibits osteogenic differentiation of hASCs in vitro.

**Fig. 3:**
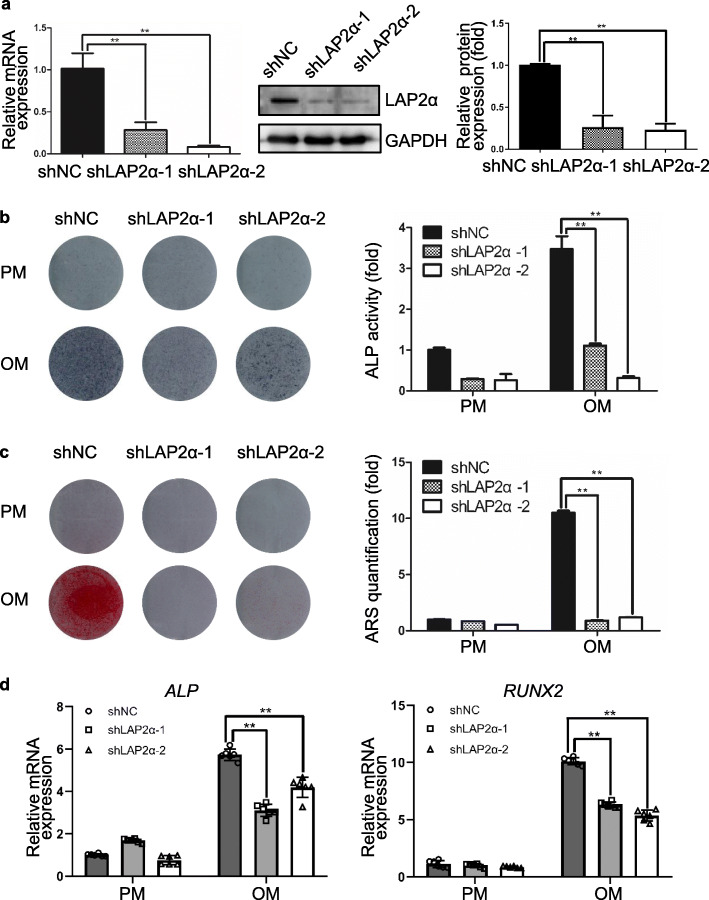
Knockdown of LAP2α suppresses osteogenic differentiation of hBMMSCs in vitro. **a** Validation of LAP2α knockdown effect by qRT-PCR and western blotting, respectively, *n* = 3. **b** ALP staining and quantification of cells in the shNC, shLAP2α-1, and shLAP2α-2 groups on day 7 after osteogenic induction, *n* = 3. **c** ARS staining and quantification of cells in the shNC, shLAP2α-1, and shLAP2α-2 groups on day 14 after osteogenic induction, *n* = 3. **d** Relative mRNA expression of *ALP* and *RUNX2* as measured using qRT-PCR on day 14 of osteogenic induction. *GAPDH* was used for normalization, *n* = 6. Data are shown as the mean ± SD; *P < 0.05 compared with the control group; **P < 0.01 compared with the control group; NS: not significant

To further test whether LAP2α regulates osteogenesis of MSCs from other sources, we established a stable LAP2α-knockdown cell line in human bone marrow-derived mesenchymal stem cells (hBMMSCs). The knockdown efficiency of hBMMSCs was assessed by qRT-PCR, western blotting and quantification (Fig. 3a). The results showed that knockdown of LAP2α significantly suppressed the osteogenic differentiation of hBMMSCs, as assessed by ALP and ARS staining and quantification (Fig. 3b, c), and qRT-PCR analysis (Fig. 3d). In summary, these results suggested that, analogous to its effect on hASCs, LAP2α is required for the osteogenic differentiation of hBMMSCs.

### LAP2α is essential for in vivo bone formation

**Fig. 4:**
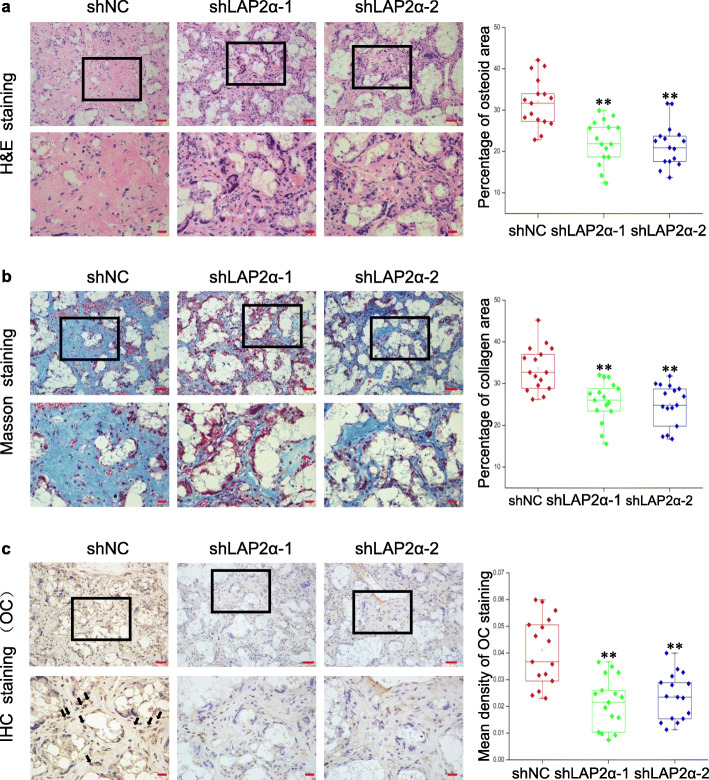
LAP2α is essential for bone formation in vivo. **a**–**c** H&E staining (**a**), Masson’s trichrome staining (**b**), and immunohistochemical staining of OCN (**c**) together with histomorphometry analysis of histological sections from implanted hASC-scaffold hybrids, *n* = 16. Dark-brown granules indicating positive staining are marked by black arrows. Low magnification images are provided in the upper panels, scale bars: 50 μm, while higher magnification images are in the lower panels (**a**–**c**), scale bars: 20 μm. Data are shown as the mean ± SD; *P < 0.05 compared with the control group; **P < 0.01 compared with the control group; NS: not significant

Next, we evaluated the effects of LAP2α on hASC-mediated bone regeneration in a xenograft model. hASCs showing stable expression of LAP2α shRNAs or the control shRNA were loaded into beta-tricalcium phosphate scaffolds and then implanted subcutaneously into nude mice. Eight weeks later, the implanted samples were excised and examined histologically. The amount of acidophilic osteoid tissue indicated by hematoxylin and eosin (H&E) staining, and collagen accumulation indicated in blue by Masson’s trichrome staining, were markedly lower in the LAP2α-deficient groups (Fig. 4a, b). Consistently, histomorphometry analysis of bone-like tissues also revealed that knockdown of LAP2α significantly reduced the area of osteoid formation (Fig. 4a, b). Furthermore, immunohistochemical staining and quantitative determination of OCN showed that the intensity and quantity of positive staining in osteoblasts were decreased in the LAP2α-deficient groups (Fig. 4c). Next, we checked the LAP2α protein expression by immunohistochemistry, and the results demonstrated that LAP2α staining signal was almost negative in tissues from LAP2α-deficient groups (Additional file 4: Fig. S4). Collectively, these results supported the view that LAP2α is necessary for hASC osteogenic differentiation in vivo.

### LAP2α knockdown activates NF-κB signaling

**Fig. 5:**
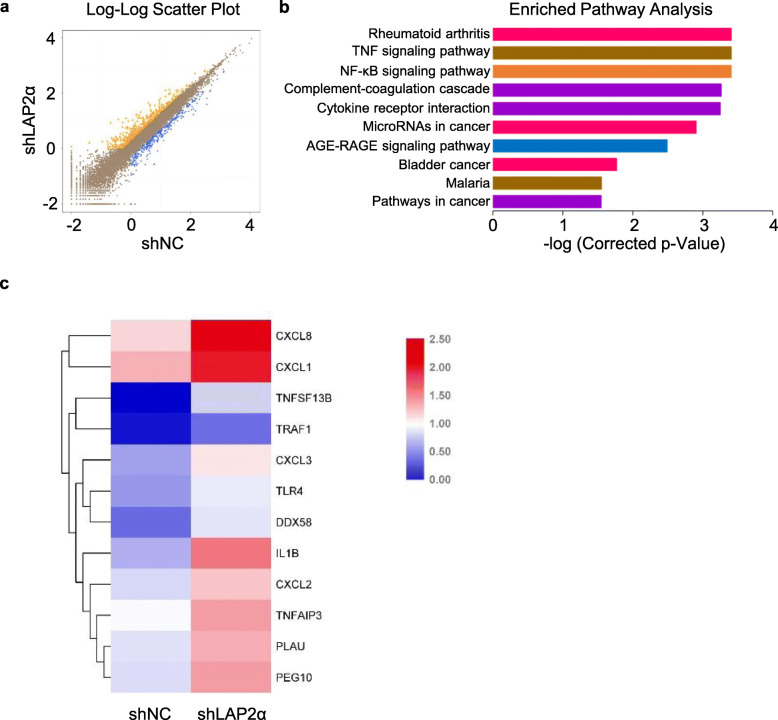
NF-κB signaling is activated by LAP2α knockdown. **a** Volcano plot showing that a total of 106 genes (yellow block) were upregulated and 91 genes (blue block) were downregulated in LAP2α knockdown hASCs. **b** Kyoto Encyclopedia of Genes and Genomes analysis showing that the differentially expressed genes were enriched in the NF-κB signaling pathway. **c** Heatmap from the RNA-seq data showing the differentially expressed genes within the NF-κB signaling pathway

To determine the molecular mechanism of LAP2α-mediated regulation of osteogenic differentiation, RNA sequencing (RNA-seq) was performed to investigate the effect of LAP2α knockdown on the transcriptome profile. The scatterplot showed that depletion of LAP2α resulted in the upregulation of 106 genes and downregulation of 91 genes (Fig. 5a). Kyoto Encyclopedia of Genes and Genomes (KEGG) pathway analysis showed that LAP2α knockdown led to changes in several important pathways, including the cell cycle, mitogen-activated protein kinase (MAPK), and NF-κB signaling pathways, which are critically related to cell growth and differentiation (Fig. 5b). Similarly, a heat map also revealed that the differentially expressed genes upon LAP2α depletion were enriched in the NF-κB signaling pathway (Fig. 5c).

**Fig. 6:**
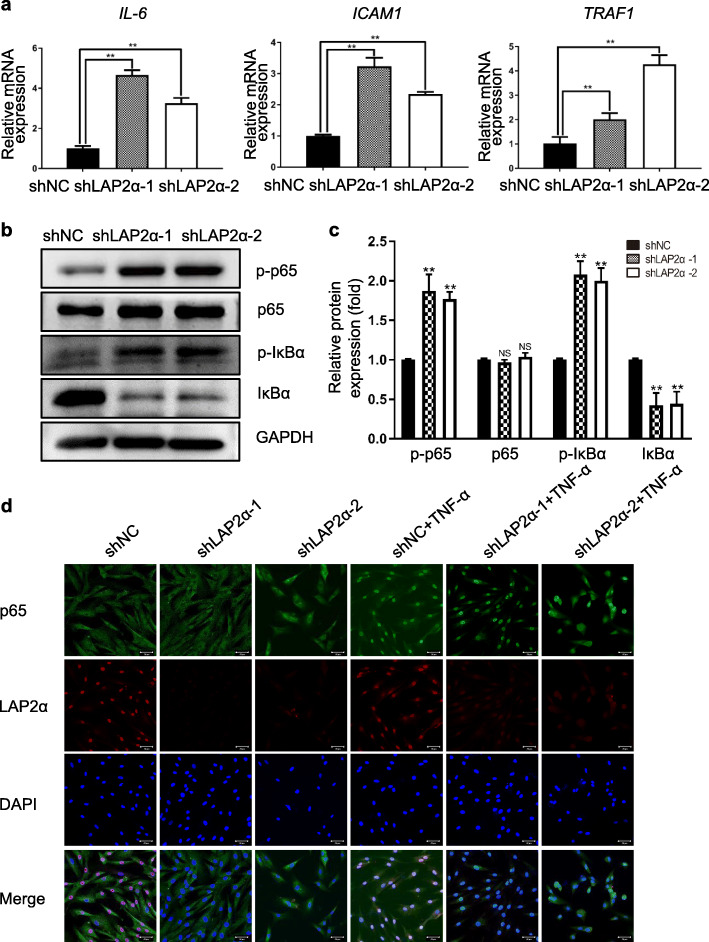
Knockdown of LAP2α promotes translocation of p65 from the cytoplasm to nucleus. **a** Relative mRNA expression levels of p65 target genes *IL-6*, *ICAM1*, and *TRAF1*, as measured by qRT-PCR, *n* = 6. **b** Proteins levels of p-IκBα, total IκBα, p-p65, and total p65 as measured by western blotting analysis in hASCs expressing shNC, shLAP2α-1, or shLAP2α-2. **c** Quantification of western blotting analysis, *n* = 3. **d** Immunofluorescent confocal microscopy of p65 nuclear translocation and LAP2α expression in hASCs expressing shNC, shLAP2α-1, or shLAP2α-2, treated or not treated with TNF-α for 30 min, scale bars: 50 μm. Data are shown as the mean ± SD; *P < 0.05 compared with the control group; **P < 0.01 compared with the control group; NS: not significant

The NF-κB pathway is considered as one of the most prominent pathways implicated in bone remodeling process; therefore, we selected certain representative genes involved in the NF-κB pathway and validated their expression levels using qRT-PCR. The results indicated that the mRNA levels of NF-κB targeted genes, such as *IL-6* (encoding interleukin 6), *ICAM1* (encoding intercellular adhesion molecule 1), and *TRAF1* (encoding TNF Receptor Associated Factor 1), were markedly increased upon knockdown of LAP2α (Fig. 6a). To understand the underlying mechanism, western blotting was conducted to check whether the classical or non-classical NF-κB signaling pathway was activated during LAP2α-mediated osteogenic differentiation of hASCs. The results showed that knockdown of LAP2α markedly increased the phosphorylation and degradation levels of IκBα and the phosphorylation of p65 (Fig. 6b, c). Consistent with the observation, immunofluorescence staining revealed that p65 nuclear translocation induced by TNF-α stimulation was enhanced in LAP2α deficient cells (Fig. 6d and Additional file 5: Fig. S5). The immunofluorescence staining also showed that LAP2α proteins were located in the nucleus. Collectively, these results suggested that knockdown of LAP2α promoted the nuclear translocation of p65 from the cytoplasm to the nucleus, which triggered NF-κB signaling.

## Discussion

In this study, we demonstrated that inhibition of LAP2α suppressed osteogenic differentiation of hASCs and hBMMSCs. Interestingly, we revealed that LAP2α-promoted osteogenesis is associated with the NF-κB pathway, as LAP2α deficiency activates NF-κB signaling in hASCs by facilitating translocation of p65 from the cytoplasm to the nucleus.

LAP2α is a chromatin-associated protein that binds A-type lamins. A series of diseases termed laminopathies are associated with mutations of A-type lamins, including Hutchinson-Gilford progeria, striated muscle diseases, neuropathies, and lipodystrophies [32, 33]. Recently, Hutchinson-Gilford progeria syndrome (HGPS) has provided an excellent model system for researchers, because HGPS shows accelerated aging as well as important bone changes that include severe osteoporosis and bone deformities [34–36]. LAP2α downregulation has been proven to be a characteristic of the HGPS cellular phenotype in previous studies [14, 15]; therefore, we hypothesized that LAP2α might play a role in MSC differentiation into osteoblasts. Indeed, we found that LAP2α expression was upregulated upon osteogenic induction. Both in vitro and in vivo experiments revealed that LAP2α deficiency resulted in impaired osteogenic differentiation of hASCs. Consistent with our observations, previous studies have reported that knockdown of A-type lamins led to impaired osteoblastogenesis and accelerated osteoclastogenesis in hBMMSCs [37, 38]. Further investigation is needed to explore whether LAP2α and A-type lamins act as a complex in the modulation of osteogenic differentiation of hMSCs.

In our in vitro study, in order to exclude the possibility that impairment of osteogenic differentiation induced by LAP2α deficiency was due to cell survival/number discrepancy, all of ALP activity and quantification of Alizarin red S staining results had been normalized to the total protein content. Moreover, a series of experiments, such as qRT-PCR and western blotting, have been performed to testify the function of LAP2α from multiple aspects. Importantly, we demonstrated that LAP2α depletion had negligible effects on the proliferation of hASCs. Therefore, the influence of LAP2α deficiency on osteogenic commitment of hASCs was not resulted from possible cell growth retardation induced by LAP2α depletion.

The present study showed, for the first time, that deficiency of LAP2α expression impairs osteoblastic differentiation of hMSCs in vivo and in vitro. These results extend a previous study in which LAP2α was reported to be critical for postnatal skeletal muscle remodeling [39]. Specifically, LAP2α absence maintained the stem cell phenotype of satellite cells, affected fiber-type determination, and delayed in vitro myoblast differentiation in murine muscle [12, 40]. Moreover, overexpression of LAP2α in fibroblasts has been shown to provoke a surge of PPARγ expression, an acknowledged key transcription factor involved in pre-adipocyte differentiation [41]. Hence, LAP2α is thought to regulate oriented differentiation of MSCs via orchestrated co-regulation together with lineage specific transcription factors.

To understand the molecular mechanism by which LAP2α regulates osteogenic differentiation of hASCs, we conducted RNA-seq to examine the effect of LAP2α knockdown on the transcriptome profile. The results showed that LAP2α-deficient cells exhibited increased expression of NF-κB-targeted genes. Furthermore, western blotting analysis revealed that deletion of LAP2α increased the phosphorylation and degradation levels of IκBα protein and the phosphorylation level of p65. For NF-κB family, the p50-p65 heterodimers remain rest in the cytoplasm through interaction with IκBα. After stimulation by TNF-α, the IκB kinase is activated, and IκBα protein is phosphorylated and degraded by the 26S proteasome [42]. Thereafter, the p50-p65 dimers are released from IκBα protein, then translocating to the nucleus [43, 44]. A growing body of evidence suggests that p65 phosphorylation plays a critical role in the accommodation of NF-κB transcriptional activity [45, 46]. Furthermore, we also observed that deficiency of LAP2α promoted nuclear translocation of p65, as verified by confocal microscopy. These results demonstrated that inhibition of LAP2α activates the classical NF-κB pathway by mediating p65 translocation. Although the intrinsic link between LAP2α and NF-κB remains to be investigated, our study provides a possible molecular network for LAP2α-promoted osteogenic differentiation of hASCs and provides a better understanding for the biological function of LAP2α.

## Conclusions

In summary, the results demonstrated that knockdown of LAP2α compromised the osteogenic differentiation of hASCs by promoting the nuclear translocation of p65, thus triggering the NF-κB pathway. These findings not only expand our understanding of LAP2α functionality, but also offer novel insights for mesenchymal stem cell-mediated bone tissue regeneration.

## Supplementary information

**Additional file 1: Figure S1.** The evaluation of transduction efficiency and LAP2α knockdown effect. a The proportion of GFP-positive cells in the shNC, shLAP2α-1, and shLAP2α-2 groups. b Protein levels of LAP2α measured by quantitative analysis of western blotting. c Validation of LAP2α knockdown effect by immunofluorescence with the indicated antibodies. Scale bars: 50 μm. * *P* < 0.05 compared with the control group; **P < 0.01 compared with the control group; NS: not significant.

**Additional file 2: Figure S2.** LAP2α knockdown has no effect on cell proliferation. a Growth curves of cells in the shNC, shLAP2α-1, and shLAP2α-2 groups determined by CCK8 assays.

**Additional file 3: Figure S3.** Microphotographs of alkaline phosphatase (ALP) staining and Alizarin red S (ARS) staining. a Microphotographs of ALP staining on day 7 after osteogenic induction. b Microphotographs of ARS staining on day 14 after osteogenic induction.

**Additional file 4: Figure S4.** Immunohistochemical staining of LAP2α. Low magnification images are provided in the upper panels, scale bars: 50 μm; while higher magnification images are in the lower panels (a-c), scale bars: 20 μm.

**Additional file 5: Figure S5.** Quantification of nuclear:cytoplasmic ratios of p65 staining in hASCs expressing shNC, shLAP2α-1, or shLAP2α-2, treated or not treated with TNF-α for 30 min. * *P* < 0.05 compared with the control group; **P < 0.01 compared with the control group; NS: not significant.

## Data Availability

The datasets used during the current study are available from the corresponding authors on reasonable request.

## Abbreviations

ALP: Alkaline phosphatase
ARS: Alizarin red S
GFP: Green fluorescent protein
hASCs: Human adipose-derived stem cells
hBMMSCs: Human bone marrow-derived mesenchymal stem cells
HE: Hematoxylin and eosin
HGPS: Hutchinson-Gilford progeria syndrome
ICAM1: Intercellular adhesion molecule 1
IL6: Interleukin 6
LAP2α: Lamina-associated polypeptide 2α
MAPK: Mitogen-activated protein kinase
MSCs: Mesenchymal stem cells
NC: Negative control
NF-κB: Nuclear factor kappa B
OCN: Osteocalcin
OM: Osteogenic media
OSX: Osterix
PM: Proliferation media
RANKL: Receptor activator for nuclear factor κB ligand
RUNX2: Runt-related transcription factor 2
TMPO: Thymopoietin
TNF-α: Tumor necrosis factor alpha
TRAF1: TNF receptor associated factor 1
