# Supplementary figures and images for: Knockdown of LAP2α inhibits osteogenic differentiation of human adipose-derived stem cells by activating NF-κB
Source: Stem Cell Res Ther. 2020 Jul 1;11:263. doi: 10.1186/s13287-020-01774-9 (PMC7329510; doi:10.1186/s13287-020-01774-9)

**Figure S1**

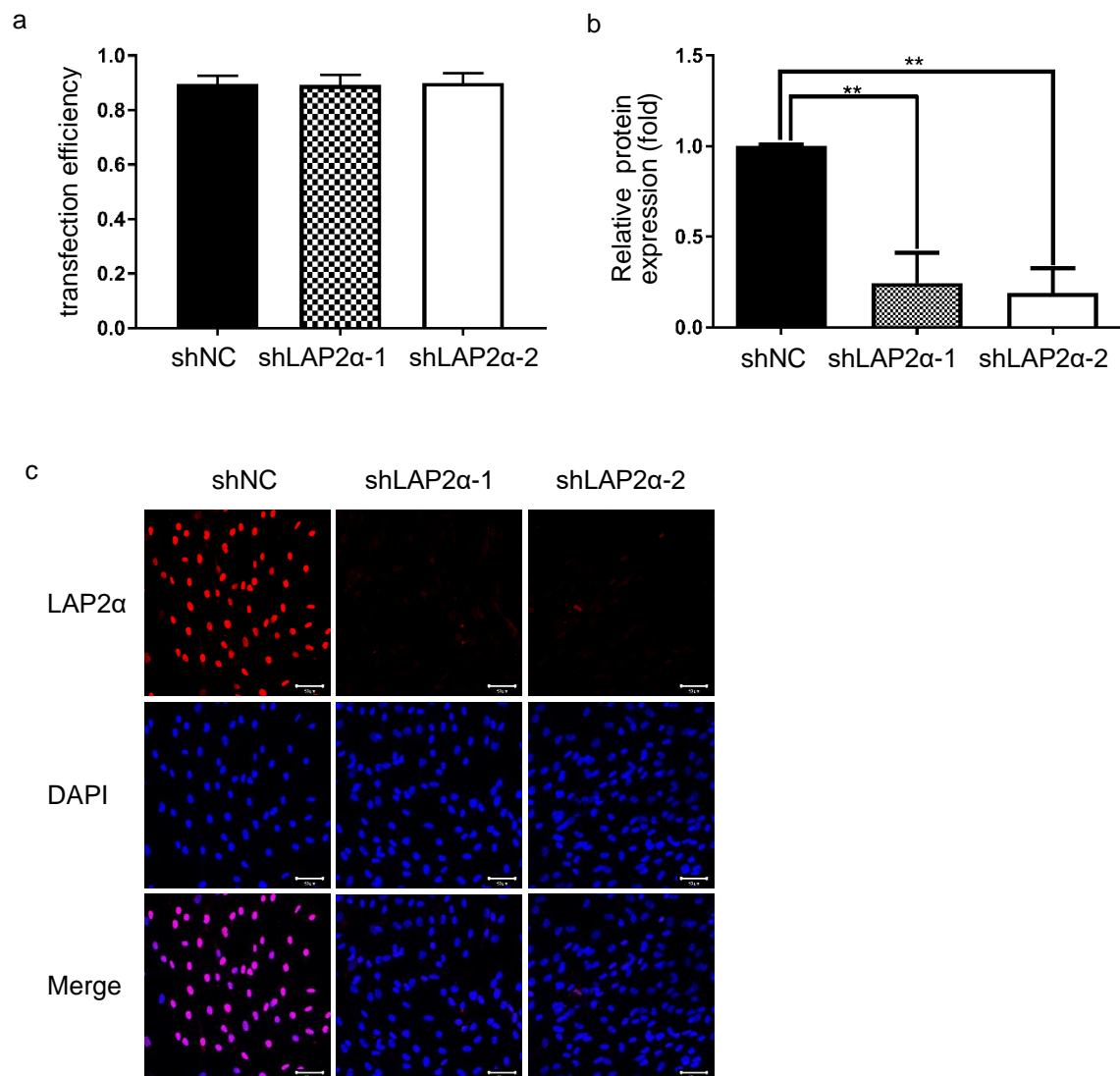

Supplement: Supplementary file 1 — Additional file 1: Figure S1. The evaluation of transduction efficiency and LAP2α knockdown effect. a The proportion of GFP-positive cells in the shNC, shLAP2α-1, and shLAP2α-2 groups. b Protein levels of LAP2α measured by quantitative analysis of western blotting. c Validation of LAP2α knockdown effect by immunofluorescence with the indicated antibodies. Scale bars: 50 μm. * P < 0.05 compared with the control group; **P < 0.01 compared with the control group; NS: not significant. [file 13287_2020_1774_MOESM1_ESM.pdf]

Figure S2

a

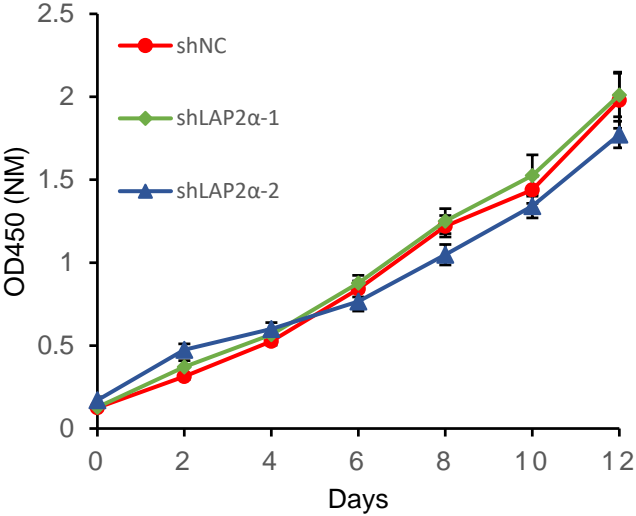

Supplement: Supplementary file 2 — Additional file 2: Figure S2. LAP2α knockdown has no effect on cell proliferation. a Growth curves of cells in the shNC, shLAP2α-1, and shLAP2α-2 groups determined by CCK8 assays. [file 13287_2020_1774_MOESM2_ESM.pdf]

Figure S3

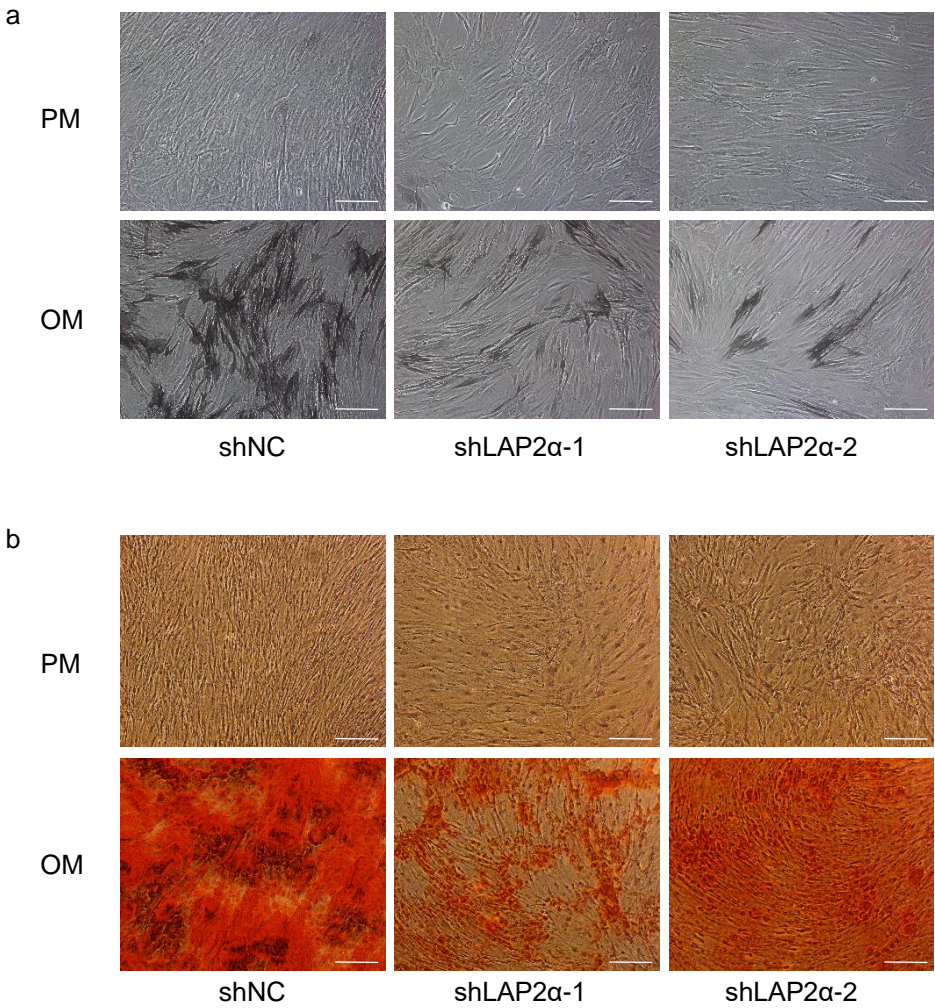

Supplement: Supplementary file 3 — Additional file 3: Figure S3. Microphotographs of alkaline phosphatase (ALP) staining and Alizarin red S (ARS) staining. a Microphotographs of ALP staining on day 7 after osteogenic induction. b Microphotographs of ARS staining on day 14 after osteogenic induction. [file 13287_2020_1774_MOESM3_ESM.pdf]

Figure S4

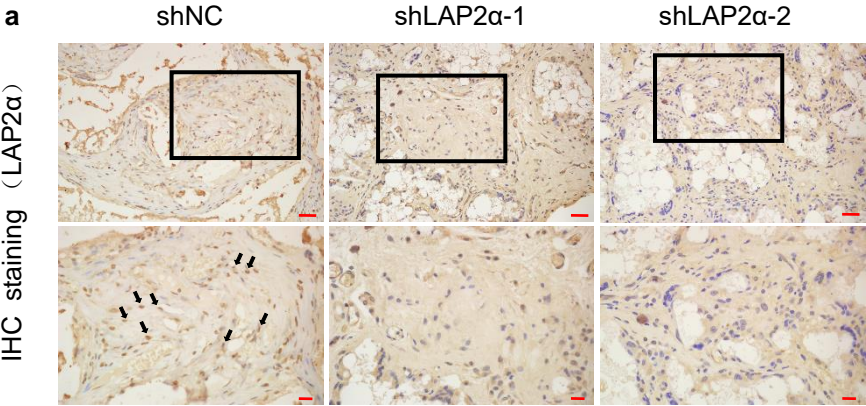

Supplement: Supplementary file 4 — Additional file 4: Figure S4. Immunohistochemical staining of LAP2α. Low magnification images are provided in the upper panels, scale bars: 50 μm; while higher magnification images are in the lower panels (a-c), scale bars: 20 μm. [file 13287_2020_1774_MOESM4_ESM.pdf]

**Figure S5**

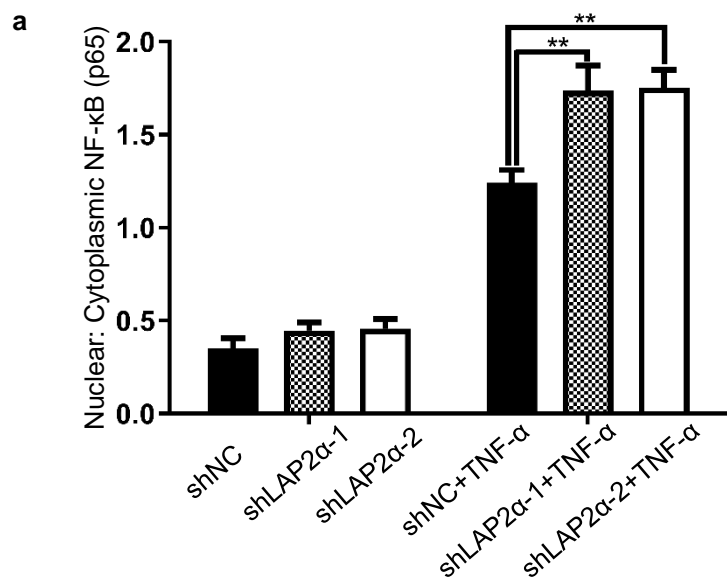

Supplement: Supplementary file 5 — Additional file 5: Figure S5. Quantification of nuclear:cytoplasmic ratios of p65 staining in hASCs expressing shNC, shLAP2α-1, or shLAP2α-2, treated or not treated with TNF-α for 30 min. * P < 0.05 compared with the control group; **P < 0.01 compared with the control group; NS: not significant. [file 13287_2020_1774_MOESM5_ESM.pdf]
